# Influence of Age on the Results of Amputation for Diseased Joints

**Published:** 1848-06

**Authors:** 


*Influence of Age on the Results of Amputations for Diseased Joints.*—In cases of amputations for diseased joints in the lower extremity, the increased mortality, after 30 years of age, is exceedingly striking; for of 32 cases of amputation performed on persons above that age, 9 perished, or one in every 3.55; whilst of 66 of those under that time of life, only 3 died, or 1 in 22. After 50 years of age the mortality becomes less than in the twenty years before that time; so that in persons between 30 and 50 years of age, the amputations for diseased joints in the lower extremity have been least successful. How carefully, then, should we in these operations consider the time of life at which our patient has arrived before pronouncing a favourable prognosis! The mortality in the second week after the operation, shows us that it is the occurrence of a less amount of secondary inflammation that the superior success of amputations in early and extreme age consists.—*Dr. Fenwick, Edinb. Monthly Journal*, 1848.

